# Ensemble perception of color in autistic adults

**DOI:** 10.1002/aur.1725

**Published:** 2016-11-22

**Authors:** John Maule, Kirstie Stanworth, Elizabeth Pellicano, Anna Franklin

**Affiliations:** ^1^ Sussex Colour Group, School of Psychology, Pevensey 1, North‐South RoadUniversity of SussexBrightonBN1 9QHUK; ^2^ UCL Institute of EducationUniversity College London55‐59 Gordon SquareWC1H 0NULondon

**Keywords:** autism, color, ensemble perception, visual perception, priors, global processing

## Abstract

Dominant accounts of visual processing in autism posit that autistic individuals have an enhanced access to details of scenes [e.g., weak central coherence] which is reflected in a general bias toward local processing. Furthermore, the attenuated priors account of autism predicts that the updating and use of summary representations is reduced in autism. Ensemble perception describes the extraction of global summary statistics of a visual feature from a heterogeneous set (e.g., of faces, sizes, colors), often in the absence of local item representation. The present study investigated ensemble perception in autistic adults using a rapidly presented (500 msec) ensemble of four, eight, or sixteen elements representing four different colors. We predicted that autistic individuals would be less accurate when averaging the ensembles, but more accurate in recognizing individual ensemble colors. The results were consistent with the predictions. Averaging was impaired in autism, but only when ensembles contained four elements. Ensembles of eight or sixteen elements were averaged equally accurately across groups. The autistic group also showed a corresponding advantage in rejecting colors that were not originally seen in the ensemble. The results demonstrate the local processing bias in autism, but also suggest that the global perceptual averaging mechanism may be compromised under some conditions. The theoretical implications of the findings and future avenues for research on summary statistics in autism are discussed. ***Autism Res** 2017, 10: 839–851*. © 2016 The Authors Autism Research published by Wiley Periodicals, Inc. on behalf of International Society for Autism Research

## Introduction

Sensory atypicalities, such as hyper‐ and hypo‐reactivity and differences in the processing of sensory information are increasingly recognized as being associated with autism [Pellicano, 2013; Rogers & Ozonoff, 2005]. These atypicalities have recently come to the fore with their inclusion in the revised diagnostic criteria for autism [American Psychiatric Association, 2013], implying that they are hallmarks of autism.

Atypical visual processing has received particular attention in the literature [for reviews, see Dakin & Frith, 2005; Simmons et al., 2009] and has led to the generation of various influential accounts of autistic perception. The weak central coherence account [Frith & Happé, 1994] posits that autistic individuals have superior access to local detail, but at the expense of the ability to extract the global gist or see “the big picture.” Several studies have shown advantages for autistic individuals in visual tasks supported by attention to local detail over global [e.g., Shah & Frith, 1983, 1993], advantages in visual search [e.g., Plaisted, O'Riordan, & Baron‐Cohen, 1998], and enhanced low‐level discrimination [e.g., Mottron, Dawson, Soulieres, Hubert, & Burack, 2006]. However, disadvantages specifically in global processing have been less forthcoming, and evidence is mixed [for a review, see Happé & Frith, 2006]. A more recent review and meta‐analysis concluded that there does not appear to be support for an overall deficit in global processing in autism, but that there is evidence for a difference in the speed with which local and global processing occurs in autism, compared to typical individuals [Van der Hallen, Evers, Brewaeys, Van den Noortgate, & Wagemans, 2015].

Pellicano and Burr's [2012] Bayesian account of sensory differences in autism builds on the central tenets of weak central coherence by situating it within a computational framework. In Bayesian models of perception, the observer is assumed to combine sensory information with a distribution of prior expectations, based on past experience. The updating of so‐called priors is reliant on the integration of visual information from current and recent experiences with past experience. Pellicano and Burr [2012] suggest that the autistic individuals have an attenuated ability to establish, maintain, and/or use priors to inform their current perception. Consequently, the distribution of prior expectations is relatively flat (i.e., has greater variance) compared to that of typical individuals. The attenuated priors account has been followed by other predictive coding theories of autism [e.g., Lawson, Rees, & Friston, 2014; Sinha et al., 2014; van Boxtel & Lu, 2013; Van de Cruys, de‐Wit, Evers, Boets, & Wagemans, 2013].

Some support for the account has been found in evidence for attenuated adaptation to faces in autistic children [Ewing, Leach, Pellicano, Jeffery, & Rhodes, 2013; Ewing, Pellicano, & Rhodes, 2013; Fiorentini, Gray, Rhodes, Jeffery, & Pellicano, 2012; Rhodes, Pellicano, Jeffery, & Burr, 2007]. Face adaptation is thought to be the result of norm‐based coding, in which the visual diet of faces to which an observer is exposed is integrated into a continually updated average face against which new exemplars can be compared [e.g., Webster & MacLeod, 2011]. The attenuated‐priors account also makes other predictions about autistic perception. For example, the ability to integrate information from a large number of sources may be crucial to the formation and maintenance of priors across the visual domain [Pellicano & Burr, 2012]. A prior is a kind of summary representation, extracted from recent experiences and representing recent instances of stimuli experienced. Summary statistics may also be extracted from a scene simultaneously (i.e., across spatial instances), whereby the features of local elements are combined and summarized to represent the global set. The attenuated priors account predicts that autistic people may be relatively weaker at extracting summary statistics from scenes [Pellicano & Burr, 2012]—a prediction that the current study seeks to test in the context of color.

Ensemble perception describes the rapid extraction of summary statistics from a set containing items which vary along some stimulus dimension [Haberman & Whitney, 2012]. Ensemble perception has been demonstrated for many different visual domains, including position [e.g., Morgan & Glennerster, 1991], size [e.g., Ariely, 2001], orientation [e.g., Parkes, Lund, Angelucci, Solomon, & Morgan, 2001], facial expression [e.g., Haberman & Whitney, 2009], facial identity [e.g., de Fockert & Wolfenstein, 2009], brightness [e.g., Bauer, 2009], and hue [e.g., Maule & Franklin, 2015, 2016; Maule, Witzel, & Franklin, 2014; Webster, Kay, & Webster, 2014]. In many of these studies, the ability to extract the average appears to exceed the limited capacity of visual working memory for representing individual items [Alvarez, 2011]. This has led to the suggestion that the extraction of summary statistics takes place in the absence of individual item representation, and requires holistic, parallel processing with attention distributed across the whole ensemble [e.g., Allik, Toom, Raidvee, Averin, & Kreegipuu, 2014; but see Myczek & Simons, 2008].

Rhodes and colleagues found that autistic children and adolescents showed differences in ensemble perception [Rhodes, Neumann, Ewing, & Palermo, 2014]—consistent with Pellicano and Burr's [2012] predictions. Participants were presented with an ensemble of four different faces (for 2,000 msec), followed by a test face which the participant had to decide whether they thought the face was in the initial ensemble. While typical participants tended to endorse a morphed mean face as part of the set, autistic participants did not. The false‐positive familiarity of the mean face in typical individuals is thought to arise from automatic extraction of the mean face—which occurred to a lesser extent in the autistic individuals.

Rhodes et al.'s procedure is, however, somewhat unusual for studies of ensemble perception in using a relatively long exposure time of 2,000 msec and a relatively small ensemble of just four faces. Previous studies of face averaging have used presentation times as low as 250 msec and sets of up to 12 faces [e.g., Haberman & Whitney, 2010]. Rapid presentation and large set sizes help reduce the possibility that serial processing of individual items is responsible for subsequent judgments about the set or about test items [Alvarez & Oliva, 2009]. Furthermore, variations in set size may be able to help establish whether judgments could be based on a small subsample of items rather than the whole set [Ariely, 2001], since an average based on a fixed subsample should become increasingly inaccurate with larger set sizes [Ariely, 2008]. The small sets and long presentation time may mean that the averaging mechanism is not required to encode the group. It is also known from adaptation studies that face coding is atypical in autism [e.g., Pellicano, Jeffery, Burr, & Rhodes, 2007; Rutherford, Troubridge, & Walsh, 2012]. Likewise, autistic individuals show difficulties in emotion, gender, identity, and gaze discrimination [for a review, see Behrmann, Thomas, & Humphreys, 2006]. Thus, one key question is whether the results of the Rhodes et al. study are specific to faces or whether they extend to other, nonface stimuli, reflecting a general property of autistic perception. To investigate this issue, it is necessary to investigate other domains of visual processing, using tasks that present larger sets in a shorter time to reduce the extent to which the serial representation of individual elements could influence the responses.

The present study investigated ensemble perception of color in autistic and typical adults. Other aspects of color perception have been investigated in autism, with varying results [Cranwell, Pearce, Loveridge, & Hurlbert, 2015; Franklin, Sowden, Burley, Notman, & Alder, 2008; Franklin et al., 2010; Koh, Milne, & Dobkins, 2010; Ludlow, Heaton, Hill, & Franklin, 2014; Maule, Stanworth, Pellicano, & Franklin, 2016]. The appearance of any particular colored surface is determined not only by the light it is reflecting, but also by the adaptation state, or “white point,” of the observer (among other factors). The white point can be understood as a statistical summary of recent visual conditions, approximating the color of the illuminant, and may play a role in color constancy [see Smithson, 2005, for a review]. Thus, the subjective appearance of a color can be understood partly by its relationship to the white point—just as adapting to a happy face makes a neutral face appear sad, adapting to a greenish illumination, for instance, would cause an objectively achromatic (i.e., white) light to appear reddish. This similarity between the norm‐based coding of faces and that of color has been noted previously [e.g., Webster, 2011; Webster & Leonard, 2008]. There is also evidence that observers show adaptation aftereffects to summary statistics—both the mean [size—Corbett, Wurnitsch, Schwartz, & Whitney, 2012] and also the variance [orientation—Norman, Heywood, & Kentridge, 2015; spatial position/numerosity—Payzan‐LeNestour, Balleine, Berrada, & Pearson, 2016]. Such aftereffects suggest that ensemble summary statistics are explicitly represented within the visual system, and may be driven by, and/or contribute to, norm‐based or relative systems of coding. Since norm‐based coding relies upon the maintenance of a neutral adaptation point, based on a summary statistical analysis of the environment, it is possible that ensemble perception might also be affected by difficulties forming and maintaining perceptual priors [Pellicano & Burr, 2012].

Various empirical studies have suggested that the influence of statistical summaries and prior experiences on current perception might be reduced in autism. In addition to the earlier‐mentioned effects of autism on face adaptation, autistic children also show reduced adaptation aftereffects for numerosity [Turi et al., 2015]. Ropar and Mitchell [2002] demonstrated that, given an unrestricted preview of an elliptical shape, autistic childen, and adolescents did not appear to weight this prior knowledge as highly as typically developing children in their estimation of the shape following a subsequent fixed point‐of‐view presentation. Autistic children also show a reduced central tendency effect when reproducing time intervals—a finding which may be accounted for by a Bayesian account of integrating prior experiences with current sensory information [Karaminis et al., 2016]. Similarly, autistic adults appear to use prior information less efficiently than typical adults in making spatial judgments about the source of sounds [Skewes & Gebauer, 2016], and in the social domain it has been shown that the integration of social cues is correlated with the extent of autism‐like symptoms in a group of typical adults [Sevgi, Diaconescu, Tittgemeyer, & Schilbach, 2016]. The predictions of the attenuated priors account do not seem to generalise to all stimuli or paradigms, however, as a number of other studies have also found null effects with regard to, for example, color adaptation [Maule et al., 2016], statistical learning [Manning, Kilner, Neil, Karaminis, & Pellicano, 2016], and adaptation to perceptual causality [Karaminis et al., 2015].

In summary, the updating and integration of prior information influencing current perception is reduced in autism, at least for some stimuli. This may be due to a deficit in extracting summary statistical information, a deficit in integrating sensory representations, or both. Ensemble perception involves the extraction of summary statistics from stimuli presented across spatial (and occasionally temporal) instances. Such summary statistics can be subject to adaptation aftereffects, suggesting that they are coded explicitly by the visual system in a fashion similar to the norm‐based or relative coding of individual stimuli such as faces and colors. Norm‐based coding, forming a prior and integrating sensory information (all of which appear to proceed somewhat differently in autism) can be understood to be summary statistical processes, the operations behind which may also be shared by the mechanism behind the extraction of summary statistics. If so, we expect that ensemble perception would be found to be different in autism.

In the present study, we used color as a substrate to investigate the representation of visual ensembles. We sought to replicate the paradigms typically used in the ensemble perception literature by using a shorter exposure time (500 msec). We also included variation in the number of elements in ensembles (four, eight, and sixteen) using two different tasks, including: (a) a membership identification task [Maule et al., 2014] providing an indication of local knowledge of individual items in the set, and (b) an averaging task [Maule & Franklin, 2015] providing an indication of knowledge of the global gist from making an explicit judgment about the mean color.

We predicted that autistic adults would show superior performance on the membership identification task, demonstrating better recognition of individual colors from ensembles than typical adults, reflecting better representation of local detail. We also predicted that autistic adults would show worse performance on the averaging task, selecting an accurate mean color to represent the mean of the ensemble less often than typical adults, representing the difficulties in extracting summary statistics predicted by the attenuated priors account.

## Method

### Participants

Twenty‐one adults (11 males) with an autism spectrum disorder (ASD) took part. All were recruited through two local autism charities to which only individuals with an independent clinical diagnosis of autism (*n* = 9) or Asperger's syndrome (*n* = 12) may be referred. Three participants who did not meet cut‐off criteria on at least one of the adult Social Responsiveness Scale II [SRS‐II; Constantino & Gruber, 2012] (*T*‐score ≥ 60) or the Adult Autism Quotient (AQ) [Baron‐Cohen, Wheelwright, Skinner, Martin, & Clubley, 2001] (score ≥ 30) were excluded from analysis. Another participant was excluded due to a particularly low IQ score (72 on Wechsler Abbreviated Scale of Intelligence—Second Edition [WASI‐II; Wechsler & Psychological Corporation, 2011] and another due to a fault during the testing session. A final sample of 16 adults (six male) formed the ASD group. The gender ratio in this sample is somewhat unusual, given the male preponderance in diagnosed cases of autism.

Twenty‐one typical adults were recruited from community contacts. Data from one participant were excluded due to a fault during testing and another did not complete the WASI‐II. Two further participants were excluded to match the ASD group in terms of mean IQ, mean age, and gender proportion (see Table 1). A final sample of 16 adults (six male) formed the typical group.

**Table 1:** Descriptive Statistics for Each Participant Group

	Group				
	Autistic adults		Typical adults		
Measure	Mean (SD)	Range	Mean (SD)	Range	Group difference
Age (years)	24.9 (4.4)	19–34	24.5 (4.2)	19–33	*t*(30) = 0.25, *P* = .807
IQ^a^	105.5 (13.7)	82–133	111.3 (10.7)	94–131	*t*(30) = 1.32, *P* = .195
AQ^b^	38.6 (5.6)	29–49	15.8 (5.8)	7–28	*t*(30) = 11.24, *P* < .001
SRS‐II^c^	78.8 (6.45)	68–90	50.0 (9.0)	26–62	*t*(30) = 10.44, *P* < .001

*Notes*: ^a^IQ, intelligence quotient, as measured by the Wechsler Abbreviated Scale of Intelligence‐II (WASI‐II; Wechsler & Psychological Corporation, 2011).

a ^b^AQ, adult autism quotient (Baron‐Cohen et al., 2001).

b ^c^SRS‐II, adult social responsiveness scale 2 (Constantino & Gruber, 2012).

All reported normal or corrected‐to‐normal visual acuity and were assessed as having normal color vision using Ishihara plates [Ishihara, 1973] and the Lanthony tritan test [Lanthony, 1998]. It was not deemed necessary to assess visual acuity objectively as the stimuli are easily visible. Participants were paid £7.50 per hour. The research protocol was approved by the local university ethics committee.

### Stimuli

Colored stimuli were chosen to represent a continuous hue circle, approximately at the monitor gamut (i.e., toward the edge of the area in color space which can be displayed by the monitor) but avoiding the extreme corners. Slight variations in luminance (i.e., the “amount of light” coming from a patch of the color, which gives rise to the sensation of brightness) were allowed (see Table 2), creating a stimulus set with colors varying in all three components of color perception—hue, saturation, and lightness. Patches were selected to be darker than the background as this helped increase the color gamut available. A uniform gray background was used throughout. Color patches subtended approximately 2° of visual angle.

**Table 2:** CIE (1931) xyY Chromaticity Values for the Colors Used in the Experiment

	CIE (1931)				CIE (1931)		
Color	*X*	*y*	*Y*	Color	*x*	*y*	*Y*
Background	0.310	0.337	30.04				
**1**	0.488	0.319	14.54	**13**	0.237	0.428	13.96
**2**	0.501	0.342	14.05	**14**	0.221	0.361	13.43
**3**	0.509	0.365	13.48	**15**	0.208	0.299	12.88
**4**	0.507	0.390	12.81	**16**	0.197	0.243	12.20
**5**	0.496	0.423	12.14	**17**	0.198	0.202	11.70
**6**	0.457	0.460	11.72	**18**	0.208	0.176	11.55
**7**	0.414	0.503	11.55	**19**	0.226	0.169	11.86
**8**	0.360	0.547	11.87	**20**	0.249	0.171	12.38
**9**	0.313	0.585	12.51	**21**	0.286	0.182	13.34
**10**	0.282	0.592	13.48	**22**	0.347	0.213	14.68
**11**	0.267	0.556	14.00	**23**	0.419	0.259	15.23
**12**	0.252	0.494	14.10	**24**	0.463	0.294	14.93

*Note*: The numbering of the colors 1–24 is arbitrary, since the complete set represents a continuous hue circle.

### Apparatus

The ensemble perception tasks were completed on a 22‐inch Mitsubishi DiamondPlus 2070SB Diamondtron CRT monitor, with a resolution of 1,600 × 1,200 pixels, 24‐bit color resolution, and a refresh rate of 100 Hz. Responses were given using a button box connected through the parallel port. A ColorCal colorimeter (Cambridge Research Systems) was used to measure the monitor and calibrate the primary values for the stimulus colors. The tasks took place in a blacked‐out room, with the monitor the only source of light. A cardboard viewing tunnel lined with black felt eliminated the effect of peripheral objects and colors and a chin rest constrained viewing distance at 57 cm, ensuring consistency of the perceived size of the stimuli.

### Design

**Figure 1:**
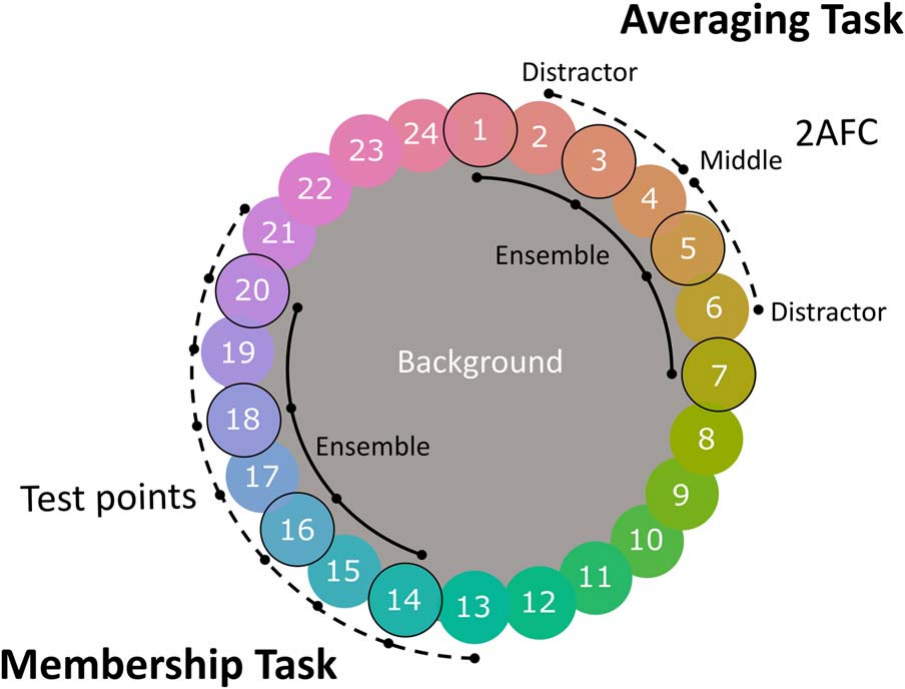
Circular arrangement of stimulus colors. The top‐right annotations indicate the arrangement of stimuli for the averaging task. The initial ensemble would contain four colors (indicated by a dark border), while the subsequent 2AFC would consist of the middle color and one of the distractors. Note that neither the middle nor the distractor color was ever present in the ensemble. The annotations to the bottom left indicate the structure of the stimuli for the membership task. Ensembles also comprised four colors but the single test point colors presented could be any of the colors spanning the ensemble range ±1. In both the averaging and membership tasks the starting point for ensembles was selected at random from this 360° circle. See online for color version. Colors rendered are an indication of those used, but are not intended to reproduce the stimuli, in print or on readers' monitors.

The experiment involved two ensemble perception tasks: (a) membership and (b) averaging. In both tasks, ensembles comprised four different colors (“members”) taken from a segment of the 24‐color stimulus circle. In terms of the color circle, ensemble members were always flanked on both sides by nonmembers (see Fig. 1) and the segment of the color circle from which the members were taken was varied at random on each trial. Ensembles contained either four, eight, or sixteen elements, resulting in three within‐participant conditions for both tasks.

**Figure 2:**
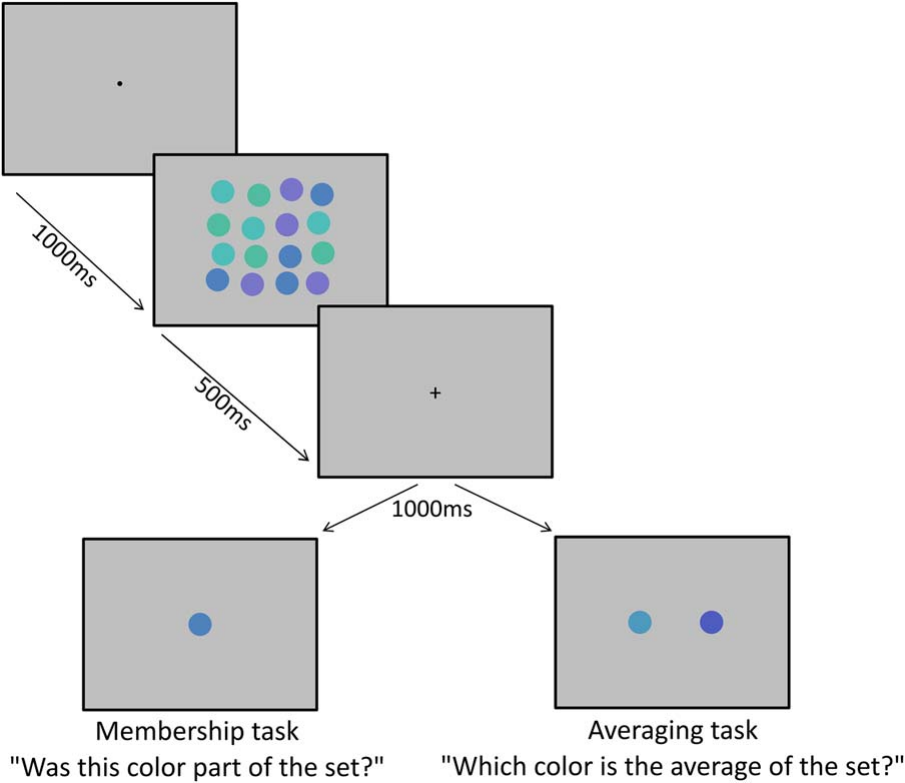
Trial procedures for the membership (left) and averaging (right) tasks. See online for color version.

In both tasks, each trial began with a black fixation point displayed for 1,000 msec. A multicolor ensemble was displayed for 500 msec, followed by a black fixation cross for 1,000 msec (Fig. 2). In the *membership task*, a single color patch was presented on the screen, until the participant responded according to whether they believed the patch was a part of the set (see Fig. 2). The button mapping (e.g., left = member, right = nonmember or vice versa) was counterbalanced across participants. The color presented could be any of the four colors from the ensemble, the three colors between the ensemble colors, or the two colors immediately adjacent to the outer colors of the ensemble (see Fig. 1). These nine conditions, multiplied by the three levels of number of elements in the initial ensemble resulted in 27 unique trial types, were completed eight times, yielding a total of 216 membership trials for each participant.

In the *averaging task*, a 2‐alternative‐forced‐choice (2AFC) display followed, with two color patches displayed. The “middle” color was always the mid‐point from the segment of the color circle from which the ensemble was generated. The “distractor” color was two color steps away from the middle, either in the clockwise or anticlockwise direction (see Fig. 1); this was counterbalanced across trials. The positions (left or right) of the middle and distractor patches were assigned at random for each trial. The 2AFC colors remained on‐screen until the participant responded by pressing a button to indicate which they thought best represented the average color. There were six types of trials, including three levels of number of elements (4, 8, 16) in combination with clockwise/anticlockwise 2AFC distractor color. Each trial type was repeated eight times per block with four blocks per participant, yielding a total of 192 trials in the averaging task. A single probe is used in the membership task to minimize the number of trials needed to provide an indication of the observer's generalization of ensemble membership [Maule et al., 2014] without the need to counter‐balance “distractors” as would be necessary using a 2AFC design.

### Procedure

Participants completed a battery of tests either in a single session lasting approximately 2 hr or in two shorter (1 hr) sessions. Order of the two experimental tasks (averaging and membership) was counterbalanced across participants. Within the session, the two experimental tasks were separated by an interval during which the participant completed the AQ [Baron‐Cohen et al., 2001] and SRS‐II [Constantino & Gruber, 2012] self‐report questionnaires. Once the second experimental task was complete, the WASI‐II [Wechsler & Psychological Corporation, 2011] was administered.

Before each experimental task, participants were briefed with instruction sheets, which explained the trial procedure and the participant's task. These instructions encouraged the participants to try to “respond as quickly and accurately” as they could. The averaging task also included an additional instruction sheet, which showed a demonstration of visually averaging a group of black lines of different lengths.

### Data Analysis

#### Membership task

Signal detection theory [e.g., Macmillan & Creelman, 1991] was used to summarize the performance of observers for the membership task. The proportion of “yes” responses to trials where the probe patch was identical to one from the preceding ensemble (member) corresponds to hits, while “yes” responses to trials where the probe did not match any color presented (nonmember) in the ensemble corresponds to false alarms. These two measures can be used to calculate *d*′ [Brophy, 1986]—a bias‐free estimate of the observers' sensitivity to the difference between ensemble members and nonmembers. Higher values of *d*′ indicate greater sensitivity to this distinction.

We also sought to establish whether there were effects of group (autistic/typical) and number of elements and whether there was any interaction between these two factors. Previous investigations have shown that color ensemble averaging is unaffected by number of elements [e.g., Maule & Franklin, 2015], suggesting that ensembles tend to be processed using global gist over local information. Given that the membership task requires attention to the local information and suppression of gist‐based representations to achieve high sensitivity, a general advantage for autistic participants may be expected. An interaction between group (autistic/typical) and number of elements would indicate that the two groups may be using local and global information differently to complete the task, since a bias toward encoding the local exemplars would result in performance declining as the number of elements increased.

#### Averaging task

The 2AFC of the averaging task design provides a bias‐free measure of detection performance, in terms of proportion of correct responses (where the observer chose the middle color from the ensemble range over the distractor). Accuracy will be examined initially in the analysis, however, accuracy is a coarse measure and does not take advantage of the variations in luminance and saturation present in the stimuli. When ensemble colors are plotted in perceptual color space (CIE L*u*v*, 1976) it becomes clear that for some ensembles the middle color is very close (i.e., similar) to the colormetric mean (defined as the Euclidean mean of the four different ensemble colors in perceptual L*u*v* color space), but for others the middle color is further away (i.e., dissimilar). There are even a small number (3 of 48) of possible ensemble‐distractor combinations in which the “distractor” color is closer to the colorimetric mean than the “middle” color. This affected, on average, only 11 trials (6% of trials) per participant.

We therefore coded each trial with the colorimetric mean of the ensemble in CIE L*u*v* space to get a better estimate of the accuracy of mean encoding and selection. Next, we calculated the three‐dimensional Euclidean distance (Δ*E*) of the chosen 2AFC color (regardless of middle/distractor status) from the ensemble's colorimetric mean. Lower values of mean Δ*E* suggest more accurate mean encoding and selection as this implies that the participant choices in the 2AFC task cluster more closely to the colorimetric mean.

#### Individual differences

To examine whether individual differences in performance was related to self‐reports of detail‐focused processing, participants' *d*′ scores on the ensemble membership task and performance on the averaging task were regressed on their scores from the “attention to detail” subscale of the AQ. Finally, a correlation was used to assess whether biases toward local or global processing result in a consistent advantage on one task and disadvantage on the other, or whether individuals appear able to adjust their focus or strategy to the task demands.

## Results

### Membership Task

**Figure 3:**
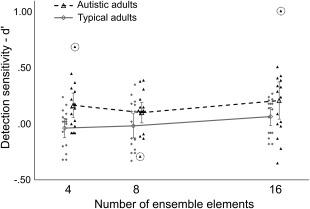
Sensitivity (*d*′) to seen and unseen test colors, by group and number of elements. Higher values of *d*′ indicate higher sensitivity. The data from the autistic group is presented as black triangles; data from the typical group as gray diamonds. Filled points represent individual performance, jittered around their *x*‐axis value for visualization purposes only. Unfilled points connected by lines represent group means for each condition. Error bars represent ±2 SEM. Dotted circles indicate data points with an absolute *z*‐score > 2 for their group and condition. These three points (*z* = 2.40, *z* = −2.13, and *z* = 2.41, left to right) belong to different observers. Removing these observers from the ANOVA has no effect on the overall interpretation of the membership task results.

The proportion of hits (responding “yes” to seen colors) and false alarms (responding “yes” to unseen colors) was transformed into *d*′ [Brophy, 1986]. A mixed ANOVA with number of ensemble elements (4, 8, or 16) as a repeated‐measures factor and group (autistic/typical) a between‐groups factor revealed that there was no main effect of number of elements (*F*(2, 60) = 1.63, *P* = .204, partial *η* ^2^ = 0.05), and no group × elements interaction (*F*(2, 60) = 0.32, *P* = .727, partial *η* ^2^ = 0.01). There was, however, a significant main effect of group (*F*(1, 30) = 11.42, *P* = .002, partial *η* ^2^ = 0.28) (Fig. 3). A follow‐up *t*‐test revealed that autistic adults were significantly more sensitive (*M* = 0.23, SD = 0.19) than the typical adults (*M* = 0.02, SD = 0.16) (*t*(30) = 3.45, *P* = .002, Cohen's *d* = 1.20).

A regression analysis of *d*′ on the “attention to detail” subscale of the AQ found that this subscale was not a significant predictor of sensitivity (*d*′) for either group (Table 3).

**Table 3:** Linear Regression of “Attention to Detail” on Task Responses

		Attention to detail (AQ)				
		*R* ^2^	*B* (SE)	*β*	*F*	*P*
Membership task (*d′*)	Typical adults	0.038	3.06 (4.10)	0.195	0.56	.468
	Autistic adults	0.004	0.62 (2.67)	0.062	0.05	.820
Averaging task (Δ*E*_4‐elem_)	Typical adults	0.032	0.11 (0.16)	0.178	0.46	.510
	Autistic adults	0.001	0.02 (0.16)	0.030	0.01	.912

*Notes*: *B*, unstandardized slope coefficient; SE, standard error; *β*, standardized slope coefficient; *n* (per group) = 16.

### Averaging Task

Responses were coded as accurate if the participant chose the color falling in the middle of the range of colors in the ensemble (see Fig. 1), rather than the distractor color from the 2AFC. Participants in both groups tended to select the middle over the distractor color, such that overall mean accuracy on the task was significantly above chance (0.5) (autistic: *M* = 0.57 (SD = 0.05), *t*(15) = 5.12, *P* < .001, Cohen's *d* = 1.28; typical: *M* = 0.58 (SD = 0.06), *t*(15) = 5.55, *P* < .001, Cohen's *d* = 1.39). There was no significant difference between the groups on overall accuracy (*t*(30) = 0.70, *P* = .490, Cohen's *d* = 0.36). A 3 (number of elements: 4, 8, 16) × 2 (group: autistic, typical) repeated‐measures ANOVA on accuracy found no main effects of number of elements (*F*(2, 60) = 0.44, *P* = .645, partial *η* ^2^ = 0.01), or group (*F*(1, 30) = 0.49, *P* = .490, partial *η* ^2^ = 0.02) and no interaction between group and number of elements (*F*(2, 60) = 2.70, *P* = .075, partial *η* ^2^ = 0.08).

**Figure 4:**
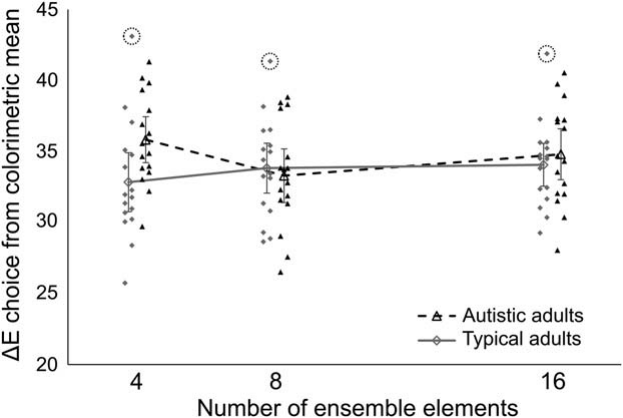
Mean distance in perceptual color space [CIE L*u*v* Euclidean distance (Δ*E*)] between the chosen color and the ensemble colorimetric mean for each group and by number of elements. Higher values indicate selections that were more perceptually distant from the colorimetric mean of the ensembles (i.e., less accurate choice of average). The data from the autistic group is presented as black triangles; data from the typical group as gray diamonds. Filled points represent individual performance, jittered around their *x*‐axis value for visualization purposes only. Unfilled points connected by lines represent group means for each condition. Error bars represent ±2 SEM. Dotted circles indicate data points with an absolute *z*‐score >2 for their group and condition. These three points (*z* = 2.47, *z* = 2.15, and *z* = 2.59, left to right) belong to the same observer. Removing this observer from the ANOVA has no effect on the overall interpretation of the averaging task results.

Raw accuracy provides a somewhat coarse indication of the observers' performance, however. The data based on perceptual difference as Euclidean distance in CIE L*u*v* space revealed a slightly different pattern of results. A 3 (number of elements: 4, 8, 16) × 2 (group: autistic, typical) repeated‐measures ANOVA on Δ*E* found no main effects of number of elements (*F*(2, 60) = 1.10, *P* = .339, partial *η* ^2^ = 0.04), or group (*F*(1, 30) = 1.10, *P* = .304, partial *η* ^2^ = 0.04). But there was a significant group × elements interaction (*F*(2, 60) = 3.83, *P* = .027, partial *η* ^2^ = 0.11). Independent *t*‐tests comparing the groups on each condition revealed no difference in the eight‐element (*t*(30) = 0.41, *P* = .684, Cohen's *d* = 0.14) and 16‐element conditions (*t*(30) = 0.63, *P* = .532, Cohen's *d* = 0.22), but the mean Δ*E* in autistic adults was significantly higher than in typical adults for the four‐element condition (*t*(30) = 2.27, *P* = .031, Cohen's *d* = 0.80) (Fig. 4).^1^ Regression of distance from the colorimetric mean (Δ*E*) for the four‐element condition on “attention to detail” (AQ) scores found that this measure was not a significant predictor of Δ*E* in either group (Table 3).

To probe whether performance on the two tasks might be related, we ran a correlation analysis between overall sensitivity in the membership task (*d*′ across all conditions) Δ*E* from the four‐element condition of the averaging task. There was no significant correlation between these measures in the typical group (*r*(14) = 0.03, *P* = .924), or in the autistic group (*r*(14) = 0.23, *P* = .390).

## Discussion

This study sought to establish whether autism is associated with reduced ability to extract summary statistics from a rapidly presented ensemble. Following the attenuated priors [Pellicano & Burr, 2012] and weak central coherence [Frith & Happé, 1994] accounts of autistic perception, we predicted that autistic adults would be less accurate when choosing the average color of an ensemble, but have an enhanced ability to remember the specific colors present in the ensemble, relative to typical adults.

The results provide some support for these hypotheses. In the membership task, autistic adults were better than typical adults at recognizing whether colors were part of the original set or not. Typical adults showed very poor sensitivity (*d*′) to the distinction between seen and unseen colors, while the autistic adults were significantly more sensitive. In the averaging task, autistic adults tended to make selections slightly further from the colorimetric mean in perceptual color space, but only in the four‐element condition. When ensembles contained 8 or 16 elements there was no significant group difference. These results suggest that the extraction and representation of average color from a rapidly presented ensemble is intact in autism in response to larger sets, but is less accurate for small sets. Despite the group differences, there was no predictive relationship found between an individual's score on the “attention to detail” subscale of the AQ and their performance on either task. Nor was there any relationship between performance on the two ensemble tasks.

This study replicates and extends that of Rhodes et al. [2014], who found reduced averaging of a set of four faces in autistic children. Furthermore, we have shown that performance on the membership and averaging tasks are not necessarily tapping the same perceptual or decision‐making processes, which means that we cannot necessarily assume that performance on a membership task is indicative of that individual's accuracy on a perceptual averaging task. Rather, the data show that a relative advantage in processing the local information of individual elements found in the autistic adults is not accompanied by any general deficit in extracting the global information of the average from each ensemble. The membership task results have implications for the interpretation of Rhodes et al.'s [2014] previous finding of reduced set averaging in children with autism. Although they used a membership task that did not directly assess the representation of the average, they found that the average face was rejected as a member of the ensemble more frequently by autistic children. In their experiment, the average was never a part of the set—it was an “unseen” face. Therefore, based on our findings, their result may be driven by better rejection of unseen items by the autism group, rather than reduced averaging per se. As Dakin and Frith [2005] point out, tasks designed to test global processing should attempt to preclude the use of local processing strategies. The membership task alone is not sufficient to make claims about extraction of global summary statistics, a task directly probing the average is also needed.

Our averaging task did precisely this. One hallmark of ensemble perception is invariance in averaging performance to changes in the number of elements in the ensemble [Ariely, 2001; Chong & Treisman, 2005a, 2005b; Haberman & Whitney, 2010; Leib et al., 2014; Marchant, Simons, & de Fockert, 2013; Maule & Franklin, 2015; Robitaille & Harris, 2011; Utochkin & Tiurina, 2014], or even improvement in averaging with larger sets [Robitaille & Harris, 2011]. Such findings are often interpreted as suggesting that rapid averaging is underpinned by a global gist‐extracting mechanism, occurring in parallel across the whole ensemble [e.g., Ariely, 2008; Treisman, 2006]. In the present study, however, there was an effect on perceptual averaging specific to small sets containing four elements for autistic adults. This may indicate that a perceptual averaging mechanism is intact in autism, at least for color, but that this mechanism is not as effectively deployed for small sets as it is in typical adults. The local processing bias in autism may cause autistic adults to apply a local strategy to small sets, but shift to a global strategy for where sets contain more items than can be represented in visual short‐term memory [Alvarez, 2011]. Further support for this view may be offered by the ideas of “Load Theory” [Remington, Swettenham, & Lavie, 2012], which suggests that autistic individuals have a greater perceptual capacity than typical individuals—leading to more visual information being processed, without attentional filtering. In ensemble tasks the encoding of more information may account for the advantage exhibited by autistic individuals in the membership task in quite a straightforward way—better encoding of the individual elements leads to better recognition. Difficulties in averaging accuracy that are selective to small sets may not be so straightforward—all of the elements are relevant to computing the set mean, and means drawn from representations of a larger sample of individual elements would, in the long run, be more accurate than one drawn from fewer individual elements. However, perceptual averaging may actually operate more accurately under conditions in which individual local element representation is minimized. Experiments manipulating local and global attention in typical adults provide some support for this idea. For example, average judgments are better when combined with a concurrent task requiring global attention, compared to a concurrent task requiring local attention [Chong & Treisman, 2005a]. Similarly, average judgments are less accurate when attention is cued locally to individual elements or when some elements are more salient than others [Albrecht & Scholl, 2010; de Fockert & Marchant, 2008]. Therefore, a bias toward local processing can explain impaired averaging performance, and furthermore, this bias is most evident in the group difference for four elements—the approximate limit for visual working memory [Alvarez, 2011].

The possibility of a shift in averaging strategy occurring around four elements in autistic but not typical observers raises the question of whether the average is computed automatically, as has previously been suggested both for size [Oriet & Brand, 2013] and location [Alvarez & Oliva, 2008]. Various studies have also shown that ensemble statistics can influence perception even when outside of attentional focus—for example, saccades to a visual target have been shown to be faster when the mean orientation of background elements is constant, compared to when the mean changes [Corbett & Melcher, 2014]. It has also been shown that responses on a categorization task are faster when a preceding prime ensemble, which does not require any response, has the same variance as the target array [Michael, de Gardelle, & Summerfield, 2014]. Tasks in which the participant is not required to respond or consider the mean or its members, but in which effects can be demonstrated, can help to indicate the automaticity of the extraction of summary statistics. If the extraction of summary statistics is atypical in autism we might expect these implicit effects to be reduced or absent, compared to a typical group. Establishing the effect of attention on summary statistical representation is a current challenge for studies of ensemble perception. Exploring perceptual averaging outside of the focus of attention may help establish whether there is a switch in strategy with larger sets, and whether this mechanism is different in autistic people.

The study is not without its limitations. First, the sample is somewhat unusual in terms of gender ratio, and represents a group of autistic adults with at least average intellectual functioning. As such the sample is rather homogeneous while the autism spectrum itself is highly heterogeneous in terms of symptoms. The lack of correlation between symptomatology (in terms of “attention to detail”) and task performance may be a further indicator of this issue. Therefore, although the results do fit well within existing frameworks and theories about autism, without testing a more diverse sample we cannot conclude definitively that reduced averaging for small sets and improved membership performance represent core parts of the autistic phenotype. Second, there are some assumptions inherent in the Euclidean distance analysis applied to the averaging task data which should be considered in the interpretation of those results. The analysis uses a particular color space (CIE L*u*v*) as an approximation of perceptual distance in order to obtain both colorimetric means and accuracy scores. This space is designed to correct some of the major nonlinearities of perception present in the CIE diagram. Although, some inequality of perceptual difference across the space remains [Witzel & Gegenfurtner, 2013], this is unlikely to result in systematic bias, and cannot account for the effect seen in the four‐element condition and not others, since the colors used are the same in each condition.

Understanding how the visual system processes simple ensembles can provide insight into how it copes with the vast amount of information it receives in the real world. Key features of autism, such as hyper‐sensitivity and sensory overload imply that the integration of information is atypical [Pellicano, 2013], while perceptual talents, such as highly accurate recall of a scene or superior visual search, demonstrate the benefits of maintaining representations of the details present in the visual world [Frith & Happé, 1994; Happé & Frith, 2006]. The present study supports the suggestion that although autism may be characterized by a cognitive style that enhances local processing, the advantages of this are not necessarily traded‐off against global processing ability [Dakin & Frith, 2005; Happé & Frith, 2006; Mottron, Burack, Iarocci, Belleville, & Enns, 2003], as is also demonstrated by the lack of relationship between performance on the membership and averaging tasks in this study. The group differences demonstrated here do not suggest a complete lack of summary representation, but do appear to reflect a difference in broad cognitive style in response to certain conditions. Our finding of typical summary representations of color for larger sets leads to further questions about whether the difficulties in the integration of visual information associated with autism reflect low‐level differences at encoding and storage [e.g., Mottron et al., 2006], high‐level differences in the integration of information [e.g., Pellicano & Burr, 2012], and/or differences in meta‐cognition [e.g., Friston, Lawson, & Frith, 2013; Lawson et al., 2014]. Similarly, the conclusions of Van der Hallen et al. [2015], that the differences in local and global processing are mainly in the speed with which such information is processed, provides further fertile ground for experimentation. If ensemble representations are a form of global processing the differences between typical and autistic observers may be amplified by shorter ensemble presentation times—reduced exposure to the ensemble may impact the encoding of the mean for autistic observers more drastically than for typical observers.

In conclusion, the pattern of responses to tasks requiring perceptual averaging and summary representation appear to be consistent with both a local bias in autism [Frith & Happé, 1994], and attenuated use of summary statistics in autism [Pellicano & Burr, 2012], but not a complete absence of their representation. The advantage for autistic adults in recognising whether they have previously seen a stimulus is not always accompanied by a disadvantage in averaging, except when sets are small. Rather, it appears that a global averaging mechanism is intact under some conditions, but that autistic adults tend to use local information by default [see Mottron et al., 2006].

## Footnotes

1 This pattern of results was consistent when the ensemble mean and distractor distances were calculated based on perceptual hue difference (angle in CIE *L***u***v** space).
